# Neisseria mucosa Urinary Tract Infection in a Neonate With Posterior Urethral Valves: A Case Report

**DOI:** 10.7759/cureus.107730

**Published:** 2026-04-26

**Authors:** Almahdi Afroukh, Asma Amarai, Taoufik Ben Houmich, Asmae Lamrani Hanchi, Nabila Soraa

**Affiliations:** 1 Department of Microbiology, Faculty of Medicine and Pharmacy of Marrakech-Cadi Ayyad University, Mohammed VI University Hospital, Marrakech, MAR; 2 Department of Biology, Laboratory of Microbiology, Mohammed VI University Hospital, Marrakech, MAR; 3 Department of Microbiology, Arrazi Hospital, Mohammed VI University Hospital, Marrakech, MAR

**Keywords:** neisseria mucosa, opportunistic pathogen, posterior urethral valves, rare infection, urinary tract infection

## Abstract

*Neisseria mucosa* is a Gram-negative diplococcus that usually colonizes the upper respiratory tract and is generally regarded as a commensal organism. It has, nevertheless, been implicated as an opportunistic pathogen in bacteremia, endocarditis, septic arthritis, meningitis, and peritoneal dialysis-associated peritonitis; urinary tract infection (UTI) remains exceptionally rare.

We report a UTI caused by *N. mucosa* in a 24-day-old male neonate with posterior urethral valves (PUVs), who presented with frank pyuria for 10 days. Direct Gram staining of urine revealed Gram-negative diplococci with a coffee-bean morphology, and the organism was recovered in pure culture. Species identification was achieved by matrix-assisted laser desorption/ionization time-of-flight mass spectrometry (MALDI-TOF MS).

Antimicrobial susceptibility testing by ETEST® showed susceptibility to ceftriaxone and resistance to benzylpenicillin and ciprofloxacin. Treatment with ceftriaxone was associated with a favorable clinical and biological outcome.

This case illustrates the opportunistic potential of *N. mucosa* in neonates with structural urinary tract abnormalities and emphasizes the value of accurate microbiological identification when UTIs occur in an unusual clinical setting.

## Introduction

Urinary tract infections (UTIs) are usually caused by Gram-negative bacilli; however, unusual pathogens may occasionally be involved, particularly in patients with underlying structural abnormalities of the urinary tract. The genus *Neisseria* comprises Gram-negative diplococci, including the strictly pathogenic species *Neisseria meningitidis* and *Neisseria gonorrhoeae*, as well as several species usually considered commensal, such as *Neisseria flavescens*, *Neisseria lactamica*, *Neisseria mucosa*, *Neisseria sicca*, and *Neisseria subflava* [1].

*N. mucosa* is a strictly aerobic Gram-negative diplococcus that typically colonizes the respiratory tract and is only rarely associated with human disease. Published reports have mainly described bacteremia, endocarditis, septic arthritis, and peritonitis [2-5]. By contrast, UTIs caused by *N. mucosa* are exceptional, with only a few pediatric cases reported to date [6,7].

Posterior urethral valves (PUVs) are a major cause of congenital lower urinary tract obstruction and predispose to UTI by promoting urinary stasis and impairing local host defenses [8]. We report a rare case of *N. mucosa* UTI in a neonate with PUV, highlighting the interplay between anatomical vulnerability and opportunistic infection.

## Case presentation

A 24-day-old male neonate, born by cesarean delivery after an uneventful, well-monitored pregnancy, had a prenatal diagnosis of bilateral ureterohydronephrosis suggestive of PUVs. He was admitted to the Pediatric Emergency Department because of frank pyuria that had persisted for 10 days.

On examination, he was afebrile (37°C), in good general condition, and had preserved urine output (1.8 mL/kg/h). There was no lumbar tenderness, no palpable bladder distension, and no systemic sign suggestive of pyelonephritis.

Postnatal ultrasound confirmed the presence of significant bilateral ureterohydronephrosis, associated with a thickened bladder wall, suggestive of obstructive uropathy due to PUVs. The diagnosis was confirmed by retrograde urethrocystography.

**Figure 1:**
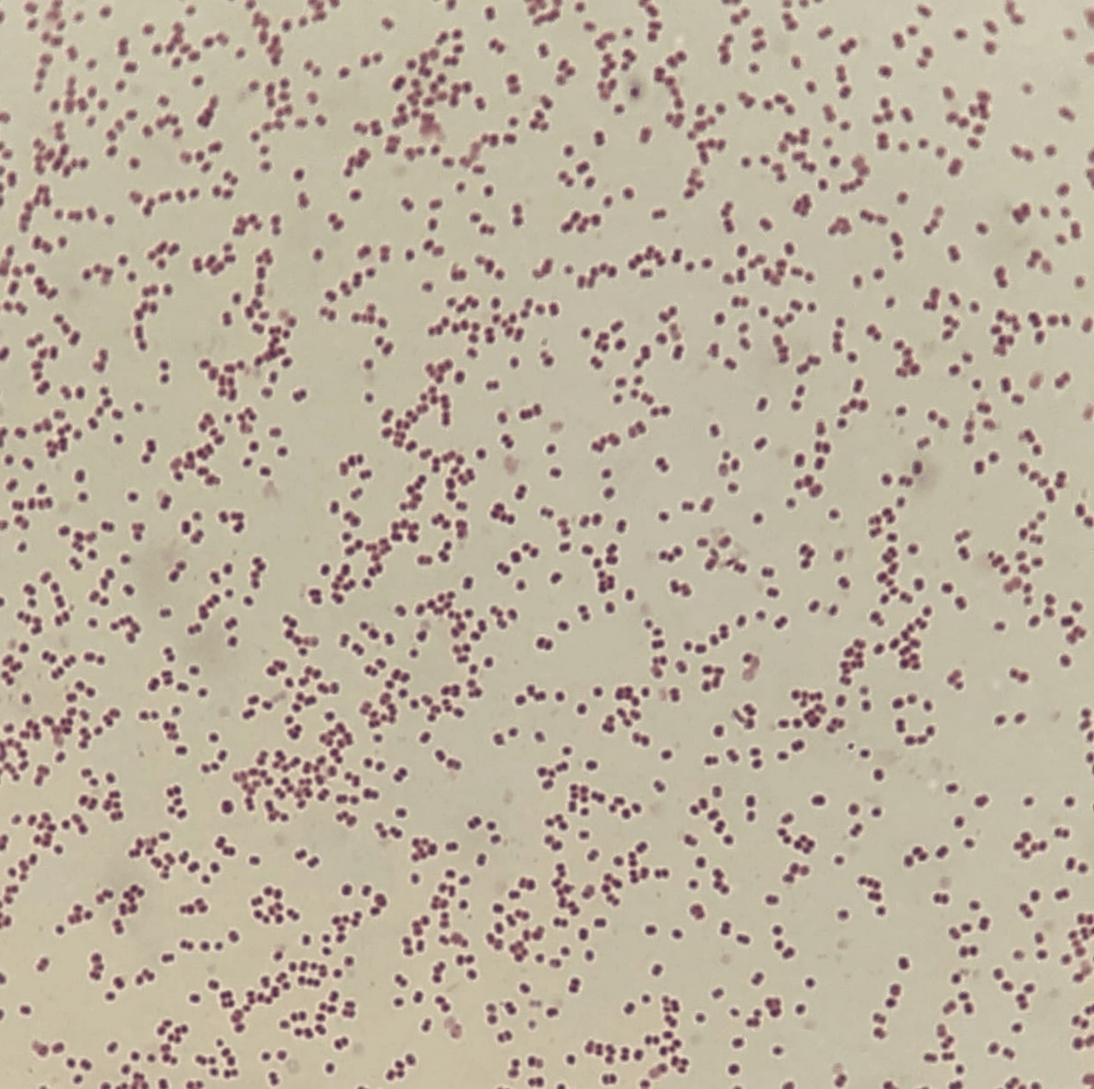
Direct Gram stain showing coffee-bean-shaped Gram-negative diplococci.

The laboratory workup showed no evidence of an inflammatory syndrome, with a normal complete blood count and C-reactive protein level. Renal function tests revealed a serum creatinine level of 49.60 µmol/L and a urea level of 5.2 mmol/L, consistent with preserved renal function for age. Cytobacteriological examination of urine, performed on a sterile bladder catheterization sample obtained under strict aseptic conditions, revealed gross pyuria, with marked leukocyturia above the diagnostic threshold (>10⁴ leukocytes/mL). Direct Gram staining showed Gram-negative diplococci with a coffee-bean morphology, suggestive of *Neisseria* species (Figure 1).

**Figure 2:**
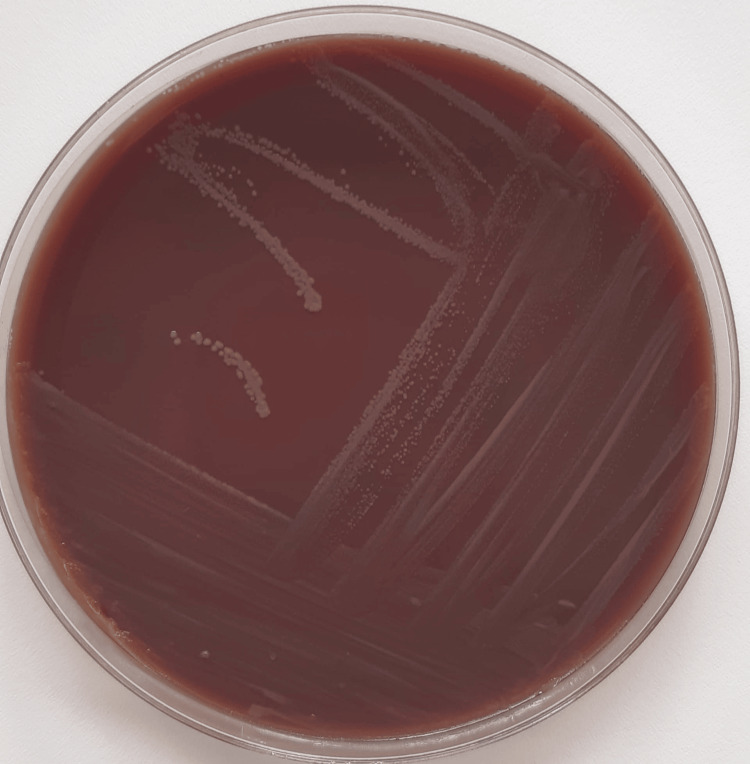
Culture of Neisseria mucosa on chocolate agar after 48 hours of incubation.

Culture on chocolate agar and blood agar yielded translucent, slightly mucoid, non-pigmented colonies after 48 hours of incubation at 35 ± 2°C in 5% CO₂. The isolate was identified as *N. mucosa *by matrix-assisted laser desorption/ionization time-of-flight mass spectrometry (MALDI-TOF MS) with a high-confidence score (Figure 2).

**Figure 3:**
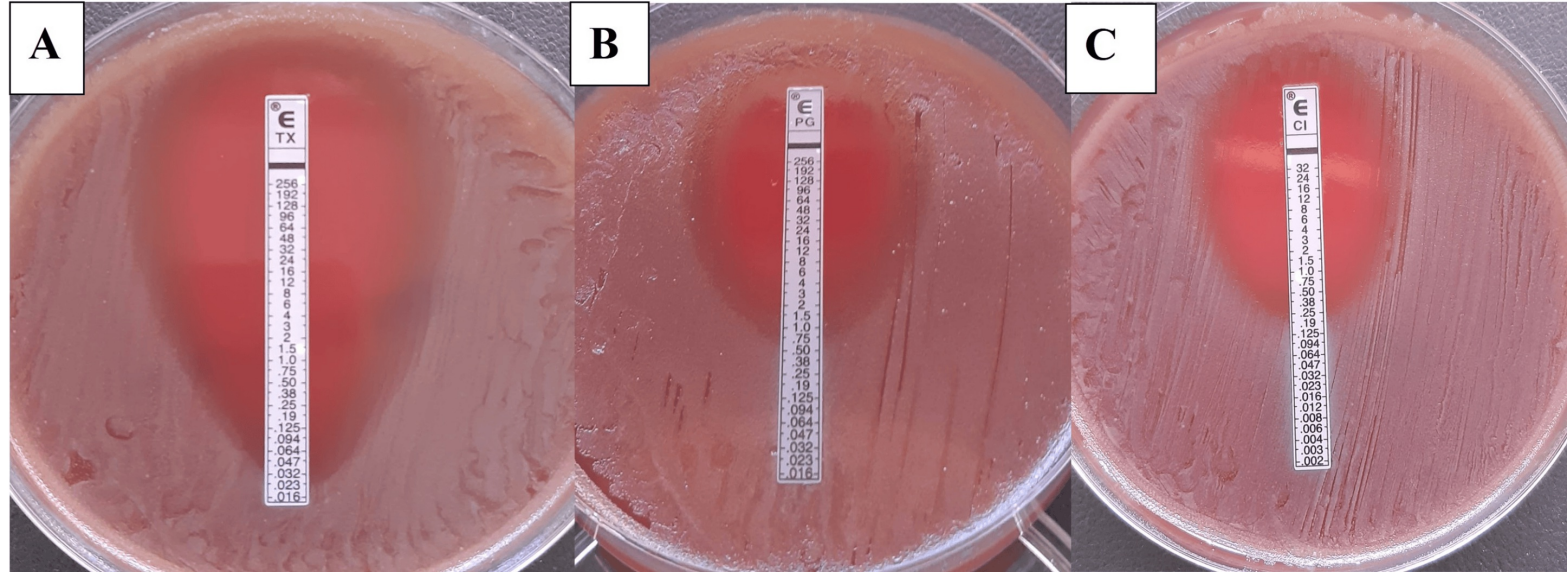
ETEST® antimicrobial susceptibility profile of Neisseria mucosa. (A) Ceftriaxone susceptibility by ETEST® (MIC, 0.47 µg/mL). (B) Benzylpenicillin susceptibility by ETEST® (MIC, 2 µg/mL). (C) Ciprofloxacin susceptibility by ETEST® (MIC, 2.5 µg/mL). MIC: Minimum Inhibitory Concentration

Antimicrobial susceptibility testing by ETEST® showed susceptibility to ceftriaxone (minimum inhibitory concentration (MIC), 0.47 µg/mL) and resistance to benzylpenicillin (MIC, 2 µg/mL) and ciprofloxacin (MIC, 2.5 µg/mL). Because no species-specific EUCAST (European Committee on Antimicrobial Susceptibility Testing) breakpoints are available for *N. mucosa*, results were interpreted using breakpoints extrapolated from *N. meningitidis* (Figure 3).

The infant was treated with intravenous ceftriaxone (50 mg/kg/day) for a 10-day course, resulting in significant clinical and biological improvement. Treatment efficacy was confirmed by a sterile follow-up urine culture, demonstrating complete eradication of the initial pathogen.

Definitive urological intervention consisted of an endoscopic incision of the PUVs, resulting in a favorable postoperative course and restoration of urinary function.

## Discussion

*N. mucosa* is a commensal species of the human nasopharynx belonging to the group of nonpathogenic *Neisseria*. It is a strictly aerobic, oxidase-positive, catalase-positive Gram-negative diplococcus. Nitrate reduction and mucoid colony morphology may help distinguish it from closely related commensal species such as *N. sicca* and *N. flavescens* [9]. Although it lacks the major virulence determinants of *N. meningitidis* and *N. gonorrhoeae*, *N. mucosa* is increasingly recognized as an opportunistic pathogen, particularly in immunocompromised patients and in those with indwelling medical devices [2-5].

This observation is noteworthy because urinary involvement by *N. mucosa* is exceedingly rare, especially in neonates. In our patient, the most plausible mechanism was ascending infection facilitated by urinary stasis secondary to PUVs. Early acquisition of commensal *Neisseria* through close contact with caregivers may provide a mucosal reservoir from which opportunistic infection can emerge when favorable local conditions are present [10].

The congenital obstructive uropathy in this case was likely a major predisposing factor, as urinary stasis and impaired local defenses favor neonatal UTI. In addition, the relative immaturity of the neonatal immune system may permit infection by microorganisms with low intrinsic virulence [11].

Several findings supported the clinical significance of the isolate rather than simple contamination: frank pyuria, marked leukocyturia, a direct smear showing Gram-negative diplococci, and recovery of *N. mucosa* in pure culture. Culture on enriched media incubated in CO₂ allowed recovery of colonies compatible with the genus *Neisseria*, and MALDI-TOF MS provided reliable species-level identification. This technology is particularly valuable for recognizing rare or unexpected pathogens in routine clinical microbiology [12].

The susceptibility profile showed resistance to benzylpenicillin and ciprofloxacin but preserved susceptibility to ceftriaxone. This is consistent with published data showing heterogeneous resistance patterns among commensal *Neisseria* species, particularly to beta-lactams. In the absence of species-specific breakpoints, interpretation according to EUCAST recommendations, extrapolated from *N. meningitidis*, remains a pragmatic approach [13].

Reported *N. mucosa* infections have predominantly been invasive, such as endocarditis, bacteremia, or septic arthritis, whereas UTI remains exceptional. As in the few previously published urinary cases, a third-generation cephalosporin was effective in our patient, supporting ceftriaxone as a reasonable option when susceptibility is documented [9,14].

## Conclusions

*N. mucosa* UTI in a neonate with PUVs is a rare but instructive example of the increased infectious risk associated with congenital urinary tract abnormalities. This case demonstrates that microorganisms usually regarded as commensals may become clinically relevant pathogens in the presence of anatomical vulnerability.

Accurate microbiological identification was central to the diagnosis in this atypical presentation, and MALDI-TOF MS played a key role by enabling rapid and reliable recognition of an unusual pathogen. Such identification facilitates early therapeutic adaptation and more judicious antibiotic use. Close collaboration between clinicians and microbiologists remains essential for the optimal diagnosis and management of these rare infections.
